# Loss of Function of *OsARG* Resulted in Pepper-Shaped Husk in *Indica* Rice

**DOI:** 10.3390/life11060523

**Published:** 2021-06-03

**Authors:** Yan Zheng, Mjomba Fredrick Mwamburi, Huaqing Liu, Feng Wang

**Affiliations:** 1 College of Life Sciences, Fujian Agriculture and Forestry University, Fuzhou 350002, China; fmjomba@tum.ac.ke; 2 Fujian Key laboratory of Crop Breeding by Design, Fujian Agriculture and Forestry University, Fuzhou 350002, China; 3 Institute of Biotechnology, Fujian Academy of Agricultural Sciences, Fuzhou 350003, China; lhq@fjage.org (H.L.); wf@fjage.org (F.W.)

**Keywords:** *OsARG*, loss-of-function mutation, pepper-like husk, indica rice

## Abstract

Grain shape is one of the most important and complex traits determining the grain yield in rice. In this study, we discovered two rice mutants with defective shape spikelets, designated as *psh1-1/2* (pepper-shaped husk 1-1/2), which were both isolated from the tissue-culture-regenerated plants of *indica* cultivar Minghui 86. The two mutants showed the same mutant phenotypes, containing pepper-shaped spikelets; shorter, smaller and compact panicles; very low seed-setting rate; high percentage of split grains; and lower grain width. Genetic analysis indicated that the mutant phenotypes were controlled by a recessive gene. Gene mapping indicated that the target gene *PSH1* was located on the short arm of chromosome 4. Sequencing analysis revealed that the two mutants each had a different nonsense mutation in *OsARG*, confirming that the target gene is *OsARG*. Compared with the previously reported *OsARG* mutant *nglf-1*, *psh1-1/2* possessed some distinct mutant phenotypes, probably because of the influence of different genetic background, suggesting that *OsARG* may function differently under different genetic backgrounds.

## 1. Introduction

Rice is one of the most important food crops for more than half of the global population. Breeding ideal superior rice cultivars with improved grain shape and other agronomic traits, such as nutritional value, disease resistance, and stress tolerance, has been the goal of rice breeders for a long time. Grain shape and grain size are important traits, which influence the grain yield in rice. They are comprehensive and quantitatively inherited, characterized by a combination of grain length, grain width, grain length-to-width ratio, and grain thickness [1].

In past decades, many genes and quantitative trait loci (QTLs) for grain shape and grain size have been identified and cloned in rice [1,2,3,4]. *GS3*, which encodes a transmembrane protein consisting of four putative domains, was the first cloned QTL controlling grain length [5]. *qGL3*, encoding a putative protein phosphatase with a Kelch-like repeat domain, also regulates grain length [6]. *GW2* [7], *GW5/qSW5* [8,9], *GS5* [10], and *GW8* [11] were found as regulators of rice grain width. It has been also found that genes affecting plant hormone biosynthesis and signal transduction can also regulate grain size in rice. Some dwarf genes, like *D1/RGA1* involved in the gibberellin pathway [12], and *D2* [13] and *D11* [14] involved in brassinosteroid (BR) biosynthesis, can also regulate the grain size of rice. Besides, some genes involved in panicle development in rice, such as *SP1* [15], *qPE9-1* [16], *EP2* [17], *DEP2* [18], and *DEP3* [19], can affect grain size or shape, too. These observations suggest that the regulation of grain shape and grain size are complicated processes controlled by a number of genes.

Flower appearance is an important character, which influences the grain shape and yield of rice profoundly. Lemma and palea are the outermost layers of rice seeds, which are the unique organs found only in *Poaceae*, responsible for protecting the florets and grains from pathogens and herbivory, and supplying carbon and nitrogen for the development of kernels [20]. Therefore, it is important for rice to establish the intact lemma and palea morphology. Some researchers have focused their study on the lemma and palea development, and a number of genes have been characterized. It has been found that rice palea might be derived from the fusion of MRP (marginal region of palea) and BOP (body of palea) [21]. MRP is smooth and light-colored, while BOP is instead populated with silicified cells bearing trichomes [22]. They are controlled in different ways. *MFO1/MADS6*, which is a member of *AGL6* clade of MADS-box genes, and *CFO1/MADS32* confer vital functions in the regulation of MRP identity, but not BOP identity [23,24]. However, *DEPRESSED PALEA 1* (*DP1*) and *OsMADS15* might be involved in the regulation of BOP identity [25]. *REP1*, encoding a CYC-like TCP transcription factor and hypothesized to be the downstream of the *DP1* gene, and *PAL1* can promote the growth of BOP and suppress the differentiation of MRP [26,27]. *LHS1/OsAMDS1* has a redundant role with *MFO1* in palea identity specification by promoting MRP development [25,28]. Besides these genes specific effects on palea development, there are many other genes conferring both palea and lemma development, such as *OPB* [29], *DH1* [30], *SHO1* [31], *SHL2* [32], *SHL4* [32], *WAF1* [33], *TH1* [34], and *BH1* [35]. Taken together, these studies suggest that palea and lemma development is a complicated process regulated by various genes.

Apart from grain characteristics, spikelet fertility is one of the critical determinants of rice grain yield. For a long time, most studies have mainly focused on male or female sterility in which plants are extremely sterile. However, low fertility is a complex phenomenon and limited knowledge of its molecular mechanism is known. The spikelet fertility is developmentally regulated by the interaction of genetic factors and environmental conditions [36]. Many defects during growth, especially under environmental stress, and developmental defects in flower structure, pollen viability, anther dehiscence, flower opening, and so on, can cause low fertility in rice [36]. Li et al. [37] reported that *POLLEN TUBE BLOCKED 1* (*PTB1*) can positively regulate the rice seed-setting rate by controlling pollen tube growth. The opening of rice flowers in the early morning helps rice to avoid sterility caused by heat stress at anthesis [38]. Lodicule is an important organ for rice because it forces the flower open through its rapid swelling [39]. Lodicule swelling is promoted by high temperature (35–45 °C) and can be induced by carbon dioxide [40]. Zeng et al. [41] reported that exogenous methyl jasmonate (MeJA), a plant hormone, can induce the floret to open through its causing swelling of lodicules by stimulating the expansion of cells. *THIS1*, encoding a class III lipase, plays a fundamental role in regulating branch and spikelet fertility through regulating pollen maturation, anther dehiscence, and flower opening [36]. In summary, spikelet fertility is a complex trait regulated by various genes and needs to be studied more.

In this study, we isolated and characterized two independent mutants, *psh1-1/2*, which exhibited pepper-shaped husks with defective palea and very low spikelet fertility. We applied a bulked segregant analysis by the whole genome resequencing (BSA-seq) method to map and clone the candidate gene, and found that the mutation of gene *OsARG* was the cause of the mutant phenotype. Compared with the published mutant *nglf-1* [42], *psh1-1/2* possessed different mutation sites and showed some different phenotypes. This work allowed us to further uncover the function of *OsARG* in regulating flower shape and spikelet fertility.

## 2. Materials and Methods

### 2.1. Plant Materials and Phenotype Observation

The rice *psh1* mutant was obtained from the progeny of tissue culture of the rice (*Oryza sativa* L. ssp. *indica*) cultivar Minghui 86 (MH86). An F_2_ population was constructed by crossing *psh1* (♀) with an *indica* cultivar 93-11 (♂), followed by selfing F_1_ individuals. All rice plants were grown in a paddy field under standard growth conditions in Fuzhou city, China. At the ripening stage, plant height, tiller number, panicle length, spikelet number per panicle, number of primary and secondary branches, seed-setting rate, grain length, grain width, and 1000-grain weight were investigated. Student’s *t*-test was performed in the statistical analysis of each trait.

### 2.2. Pollen Viability Assay

To determine the pollen viability of *psh1*, anthers were collected from a spikelet at the heading stage and moved onto the glass slide. After the anthers were broken, pollens were stained with I_2_-KI staining buffer consisting of 1% I_2_ and 3% KI at room temperature. Then, 20–30 min later, the pollen grains were observed using a light microscope and photographed. MH86 was used as control.

### 2.3. Mapping of PSH1 Using BSA-seq Method

Leaves of 20 mutant plants (M-pool) and 20 wild-type plants (W-pool) were collected from the F_2_ population and bulked, respectively, for DNA extraction using the CTAB method [43]. Two paired-end DNA-sequencing libraries with an average insert size of 400 bp were constructed for the two pools using the Illumina TruSeq DNA LT kit (ID: FC-121-2001) according to the manufacturer’s instructions (Illumina, San Diego, CA, USA). Each library was sequenced on an Illumina HiSeq 2500, generating a total of 4.6 Gb data, representing an average of 12× coverage of the rice genome. The two parents, MH86 and 93-11, were used as control, and had been sequenced by Fujian Academy of Agricultural Sciences at an average depth of 50× and 30×, respectively (data unpublished). The alignment and variant calling were performed as described [44]. The biquadratic of average allele frequency difference between the two pools (denoted as AFD^4^) was calculated to detect the target gene *PSH1*. The highest peak of AFD^4^ was considered to be the target gene region.

### 2.4. Fine Mapping of PSH1

One hundred mutant plants were selected from the F_2_ population. DNA was extracted from the fresh leaves of each individual and the two parents. InDel and SNP markers were developed in the target region according to the genomic sequence data of MH86 and 93-11 and used to genotype the F_2_ plants. Primers were designed using Primer 3 (https://bioinfo.ut.ee/primer3-0.4.0/ (accessed on 24 May 2021)) and synthesized by Shanghai Sangon Biological Engineering & Technology Company (Shanghai, China). PCR was performed in a 15 μL reaction mixture containing 20–50 ng genomic DNA, 200 μM of each dNTP, 1× PCR buffer (10 mM Tris-HCl, pH 8.4, 50 mM KCl, 1.5 mM MgCl_2_), 0.5 μM each of forward and reverse primers, and 1U Taq polymerase. All reactions were run with a routing PCR program: 4 min at 94 °C, 30 cycles of 30 s at 94 °C, 30 s at 58 °C, and 1 min at 72 °C; and 5 min at 72 °C for final extension. PCR products were separated using 6% non-denaturing PAGE (300 V, 2 h) and stained with silver following the method of Xu et al. [45] with minor modifications. Linkage analysis was performed using Mapmaker EXP 3.0 [46] between the molecular markers and the *PSH1* locus. The Kosambi mapping function was used to convert recombination rate into map distance. Markers were linked with an LOD threshold of 3.0.

## 3. Results

### 3.1. psh1 Mutant Produces Pepper-Shaped Spikelet

**Figure 1:**
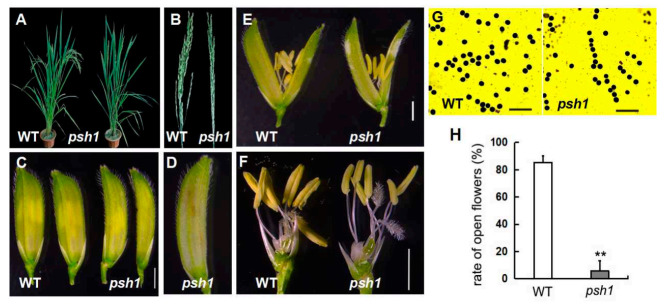
Phenotype of *psh1* mutant. (**A**) Phenotype of WT (MH86) and *psh1* plants at the heading stage. (**B**) Panicles of WT and *psh1* at the heading stage. (**C**) Spikelets of WT and *psh1*. Bar = 2 mm. (**D**) A spikelet of *psh1* showing brown inside. (**E**) Opened spikelets of WT and *psh1*. Bar = 2 mm. (**F**) Spikelets of WT and *psh1* with lemma and palea removed. Bar = 2 mm. (**G**) Pollen viability test using potassium iodide staining. Bar = 200 μm. (**H**) Rate of open flowers of WT and *psh1*. Asterisks indicate significant differences to wild type (MH86) at *p* < 0.01 (**) according to *t*-test (*n* = 10).

The phenotype of mutant *psh1* was examined at various stages of growth and development. During vegetative growth, *psh1* displayed a similar phenotype to MH86, with no apparent morphological changes. Their plant height and tiller number were similar (Table 1). At the heading stage, *psh1* produced flowers at the same time as MH86. The plant architecture of *psh1* was slightly compact compared with that of MH86 (Figure 1A). The panicle of *psh1* was ~20% shorter and more compact than MH86 (Table 1). Unlike MH86, the panicle of *psh1* was partially enclosed by the leaf sheath (Figure 1B). However, the numbers of primary and secondary branches displayed no difference between *psh1* and MH86 (Table 1). The spikelet of *psh1* showed a pepper-like shape with a slightly larger length but smaller width compared to that of MH86 (Figure 1C; Table 1). As shown in Table 1, the spikelet number per panicle of *psh1* was less than that of MH86, and the seed-setting rate of *psh1* (5.8%) was remarkably less than that of wild type (85.2%).

In order to explain the reduced fertility of *psh1*, we examined the internal structure of the *psh1* flower and found that there were no apparent distinguished changes compared to the wild type (Figure 1E,F). Pollen viability analysis showed that the size, morphology, and staining pattern of *psh1* pollen grains were similar to those of wild type (Figure 1G). However, the opening and closing of the *psh1* flower was abnormal. There were many more unopened flowers in the inflorescence of *psh1* than in the wild-type inflorescence. Only a small percentage (6.55%) of flowers eventually opened in *psh1*, compared to 84.65% in wild type under the same condition (Figure 1H). As a result, the anthers in unopened flowers became brown and inactive (Figure 1D). These observations suggested that the reduced fertility of *psh1* is not caused by floral structure and pollen grain activity, but by the behavior of the flower opening, at least in part.

### 3.2. Abnormal Grain Size and Grain Shape of psh1

**Figure 2:**
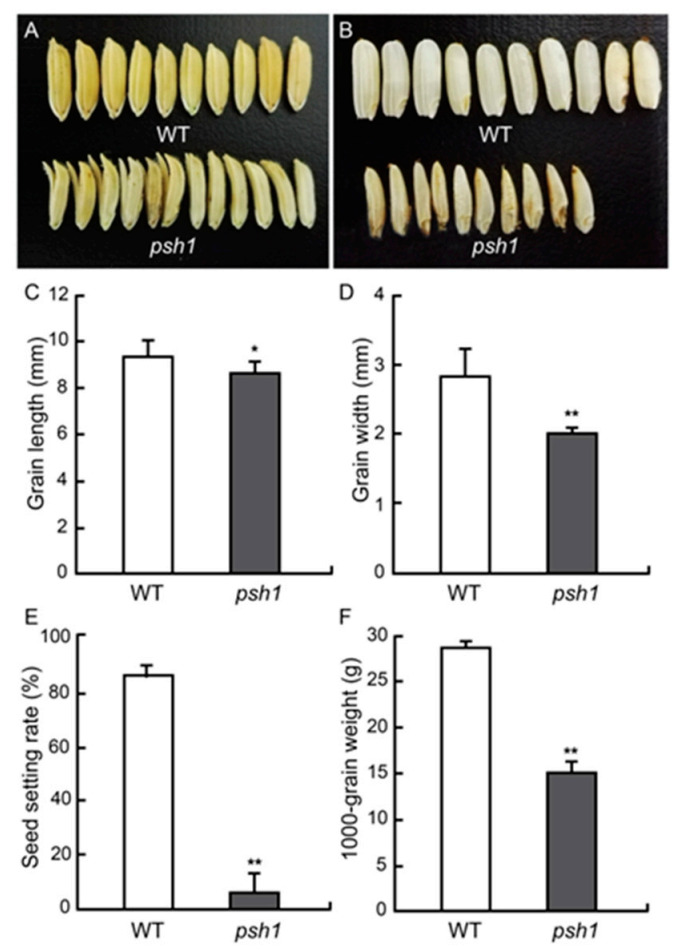
Comparison of mature grains. (**A**) Seeds of WT and *psh1*. (**B**) Dehulled grains of WT and *psh1*. (**C**) Grain length. (**D**) Grain Width. (**E**) Seed-setting rate. (**F**) Thousand kernel weight. Asterisks indicate significant differences at *p* < 0.05 (*) or *p* < 0.01 (**) according to *t*-test. Error bar represents SD. *n* = 10.

At the mature stage, the grain size and grain shape of *psh1* were analyzed. The seeds produced by *psh1* appeared smaller than those produced by wild type, and many of them (62.75 ± 0.05%) had split glumes (Figure 2A). The grain length (8.7 ± 0.48 mm) and width (2.0 ± 0.08 mm) of *psh1* were significantly less than those of wild type (Table 1; Figure 2C,D). When lemma and palea were removed, the inside rice of *psh1* was much smaller than that of wild type and showed a pepper shape (Figure 2B). As a result, the 1000-grain weight of *psh1* was reduced a lot compared with that of the wild type (Table 1; Figure 2F).

### 3.3. Genetic Analysis of psh1

F_1_ plants of the cross between mutant *psh1* and wild type 93-11 showed the wild-type phenotype, including spikelet shape and fertility, suggesting that the mutant allele of *PSH1* was recessive. In F_2_, 126 of 461 plants showed pepper-shaped seeds and low fertility. The segregation of wild-type and mutant plants fitted the ratio of 3:1 (*χ*^2^ = 1.33, *P* = 0.248). This result indicated that the mutant phenotype of pepper-shaped husk and low fertility was controlled by a single recessive gene.

### 3.4. Mapping of PSH1

**Figure 3:**
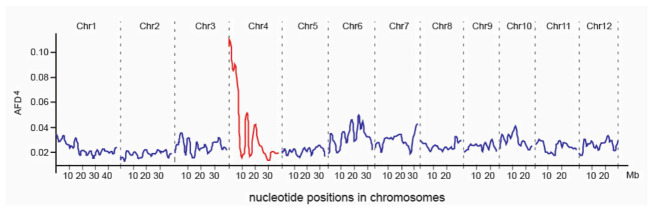
AFD^4^ profile across the rice genome. The highest AFD^4^ peak was detected on chromosome 4.

The BSA-seq approach was used to map the *PSH1* gene based on the F_2_ population. A total of 670,753 variants of DNA sequence (including 591,231 SNPs, 67,799 short InDels and 11,723 others) were identified and selected as markers for mapping *PSH1*. The highest peak of AFD^4^ was found at the beginning of the short arm of chromosome 4, covering a region of ~3 Mb (from 0 Mb to 3.0 Mb in the physical map), with the climax at ~0.3 Mb (Figure 3), which suggested that *PSH1* is likely located in this region.

**Figure 4:**
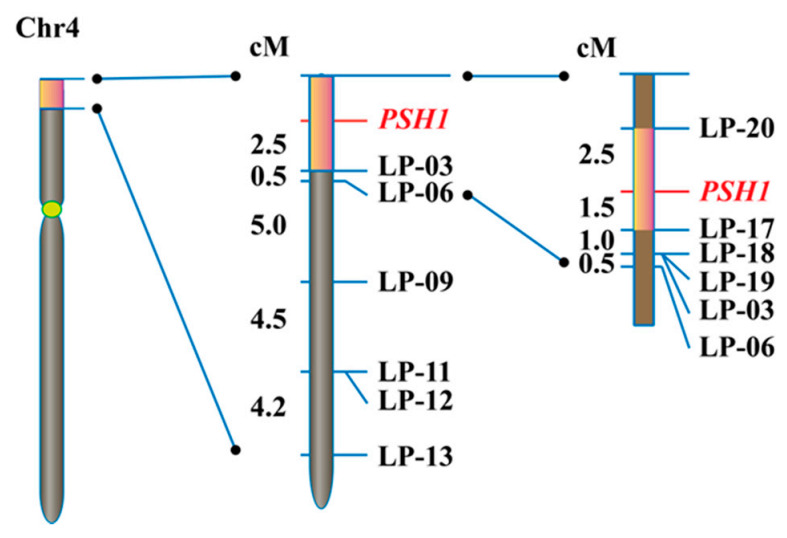
Fine mapping of *PSH1*. The orange boxes indicate the regions harboring the target gene *PSH1* on chromosome 4 determined by BSA-seq (**left**), primary mapping (**middle**), and fine mapping (**right**), respectively. The names of InDel markers and the target gene are shown on the right of the chromosome. The genetic distance (cM) between adjacent loci are shown on the left of the chromosome.

To fine map *PSH1*, a set of InDel markers were developed in the target 3 Mb region of chromosome 4 using the information from the sequencing data. One hundred plants with pepper-shaped husks and low fertility from the F_2_ population were genotyped with the InDel markers. After linkage analysis, *PSH1* was primarily mapped in a region between the start of chromosome 4 and marker LP-03, with a distance of 2.5 cM from LP-03 (Figure 4). Using markers developed in this region, *PSH1* was further mapped to a 4.0 cM or 218 kb interval delimited by markers LP-17 and LP-20 (Figure 4).

### 3.5. Identification of PSH1

**Figure 5:**
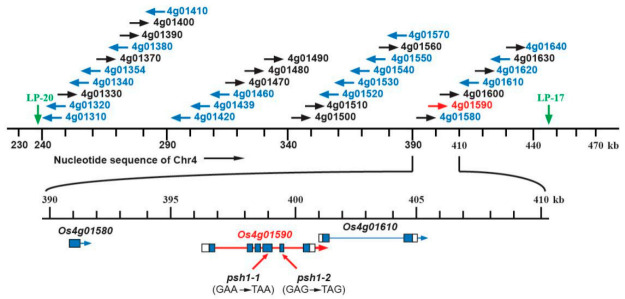
Identification of *psh1* as a nonsense mutant of *LOC_Os04g01590*. The 218 kb genomic region between LP-20 and LP-17 contains 31 coding sequences (CDSs). Two mutant alleles were found in *LOC_Os04g01590*. One contained a substitution of GAA to TAA in the fourth exon, and the other contained a substitution of GAG to TAG in the fifth exon. Both mutations resulted in a premature stop in the CDS. No nucleotide change was found in the remaining 30 CDSs. Black/blue arrows indicate the directions of CDSs opposite to/the same as that of the chromosome 4 nucleotide sequence. The red arrow highlights *LOC_Os04g1590* as the candidate gene for *PSH1*. The exon-intron structures of *LOC_Os04g1590* and its two neighboring CDSs are depicted below the chromosome 4 nucleotide sequence.

**Figure 6:**
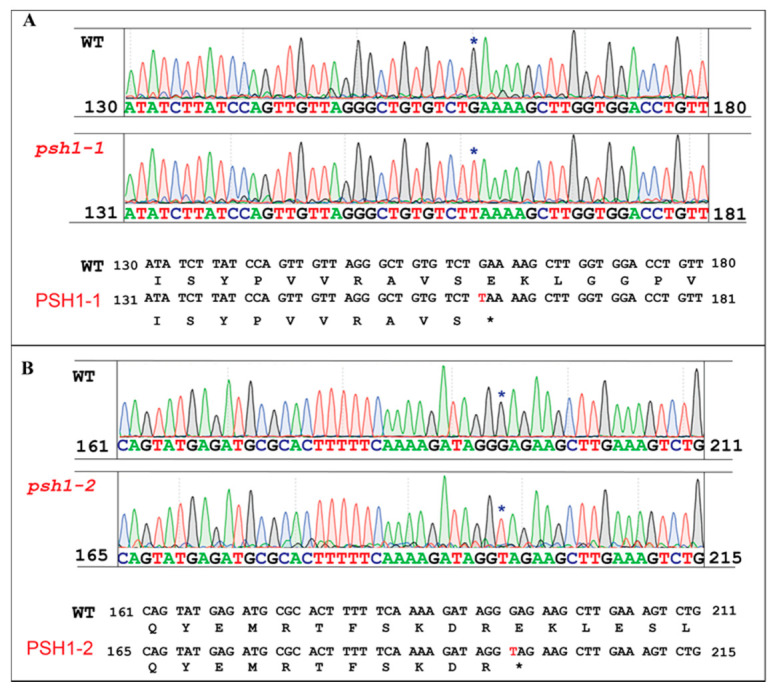
Sequence analysis of *psh1* mutant. Two independent point mutations resulting in stop codons were found in the fourth and the fifth exons of the *LOC_Os04g01590*, respectively. The two mutant alleles were named *psh1-1* (**A**) and *psh1-2* (**B**), respectively. The mutated bases and codons are marked with asterisks.

To identify the candidate gene of *PSH1*, the open reading frames (ORFs) in the region between LP-17 and LP-20 were analyzed. A total of 31 putative ORFs were found (Figure 5). We examined the target mutation site of *psh1* by comparing the assembled genomic sequences of the 31 ORFs in the W-pool, M-pool, MH86, and 93-11, based on the expectation that the M-pool would only contain the mutant allele, while the W-pool would contain the mutant allele and the wild-type (MH85 and 93-11) allele simultaneously. Using this method, a nucleotide substitution (G to T) mutation was found at the fourth exon of gene *LOC_Os04g01590*, which resulted in premature termination of the protein encoded by the gene (Figure 5). To verify the mutation, we developed a pair of primers (Loc-590-2F: TCCAGTTGTTAGGGCTGTGT and Loc-590-2R: GCCTCCTAGCATAACCTCCT) flanking the mutation site (G to T) to amplify the sequence in 40 mutant plants from the F_2_ population. After sequencing the amplified sequences, it was found that 31 mutant plants had the expected nucleotide substitution (Figure 6A). However, the other 9 mutant plants did not contain the mutation. By rechecking the sequence of *LOC_Os04g01590*, we found that there was another nucleotide substitution (G to T) occurred in the fifth exon of *LOC_Os04g01590*, which also led to a premature stop codon (Figure 6B). However, the M-pool contained the mutant allele of this site as well as the wild-type allele, and the mutant allele actually only accounted for a small proportion in the M-pool. That is why it was ignored initially. Using a pair of primers (Loc-590-5F: GTAGGATCCTCTTCGGCCAT and Loc-590-5R: TGTCCAAACAGA GCATCTACG), we verified that the 9 mutant plants did carry the mutant allele. Therefore, there were two mutant alleles of *LOC_Os04g01590* in the F_2_ population, but the second mutant allele had a lower frequency. The reason for two mutant alleles existing in the same F_2_ population might be that the two types of mutants, which could not be distinguished in morphology, were both used as parents to be crossed with 93-11. We designated the first mutant as *psh1-1* and the second mutant as *psh1-2*. The two different mutations of *LOC_Os04g01590* causing the same mutant phenotypes confirmed that *LOC_Os04g01590* is the candidate gene of *PSH1*.

### 3.6. PSH1-1/2 Are New Alleles of OsARG

**Figure 7:**
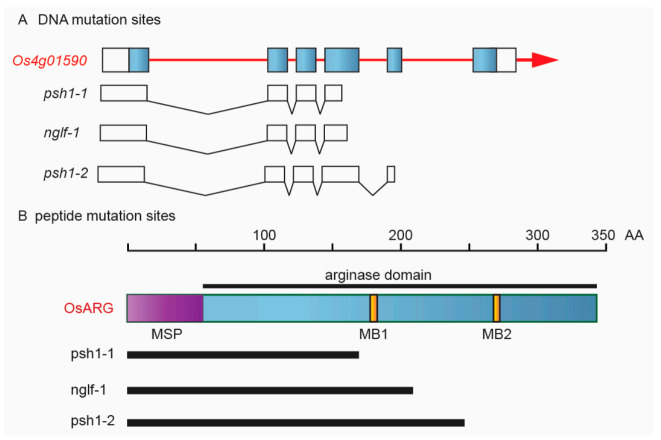
Different mutation sites of OsARG in the mutants *psh1-1*, *psh1-2*, and *nglf-1*. (**A**) DNA mutation sites of gene LOC_Os04g01590. (**B**) Peptide mutation sites of OsARG. MSP: mitochondrial signal peptide; MB: Mn^2+^ binding site.

The Rice Genome Annotation Project (http://rice.plantbiology.msu.edu/ (accessed on 24 May 2021)) identifies *LOC_Os04g01590* as a putative arginase gene. It is a rice ortholog of *Arabidopsis ARG* gene. *OsARG* has six exons and encodes a protein of 340 amino acids. OsARG contains one mitochondrial targeting peptide at the N-terminal end, a conserved arginase domain, and two Mn^2+^ binding (MB) sites (Figure 7). The stop codon mutation in the fourth exon in *psh1-1* and that in the fifth exon in *psh1-2* resulted in loss of two and one MB site, respectively. They were two new alleles of *OsARG*.

## 4. Discussion

In this study, we identified two new mutants of *OsARG* (*psh1-1/2*) caused by nonsense mutations, which both resulted in truncated OsARG peptides with one or two MB boxes deleted. Therefore, the function of *OsARG* must be completely lost in the two mutants. The two mutants had the same phenotype and were indistinguishable in morphology, both showing defective palea, pepper-shaped husks, split glumes, and low seed-setting rates. Our findings suggest that *OsARG* is an important gene with multiple effects on rice plant development.

A mutant of *OsARG* named *nglf-1* has been reported before [42]. Similar to *psh1-1/2*, *nglf-1* also shows a dramatically low seed-setting rate. However, unlike *psh1-1/2*, *nglf-1* does not show significant changes in grain shape, except for becoming a little narrower than wild type. As the mutation of *OsARG* in *nglf-1* also results in a truncated peptide with the MB2 deleted, as is the case with *psh1-2* (Figure 7), the *OsARG* function is also completely lost in *nglf-1*. Therefore, the different mutant phenotypes observed in *psh1-1/2* and *nglf-1* may probably be caused by the difference of genetic background between them. The mutant *psh1-1/2* and the F_2_ population used in this study all had the genetic background of *indica* rice. In contrast, the genetic background of *nglf-1* is more complicated. The mutant *nglf-1* is generated from the anther culture of a tetraploid *indica*/*japonica* hybrid H3774. Therefore, *nglf-1* is a mixture of *indica* and *japonica* background. It is possible that the functionless mutant alleles of *OsARG* interact with the different genetic background, resulting in different mutant phenotypes. This suggests that *OsARG* may function differently under different genetic backgrounds. 

Flower opening is an important process for seed-setting in rice. Before floret opening, the anthers will begin to dehisce when they reach the top of the floret. At the flowering time, the two lodicules become turgid and force the lemma and palea open. After floret opening, the filaments elongate further, and the remaining pollen grain is released. Then, the floret closes and keeps the empty anthers outside [36]. In spite of the premature termination of OsARG protein, the three mutants can still obtain some seeds. The seed-setting rate of *psh1-1/2* is 0.07 times that of wild type, a little lower than that of *nglf-1* (0.11 times that of wild type). We speculate that the low seed-setting rate of these mutants is caused by the defect of the flower opening. In *psh1-1/2*, we found that most florets could not open at the flowering time, so that the anthers inside the floret lost activity and became brown. In addition, for the opened florets, many could not close normally after pollinating, so that more than half of the mature seeds showed glume split. To investigate the cause of the unopened florets, we examined the size and structure of lodicules in *psh1-1/2*, but found no difference from those of wild type (Figure 1F; Figure S1). Therefore, it appears that the abnormal activity of the flower opening and closing in *psh1-1/2* has no relationship with lodicules. Further study is needed to uncover the mechanism.

*OsARG* is the only gene encoding arginase in rice, which is one of key enzymes in the Arg catabolism pathway [42]. In *nglf-1*, Arg catabolism is blocked, resulting in nitrogen shortage and the aberrant phenotype in the panicle, which can be partially recovered by exogenous nitrogen. We also performed a similar test on *psh1-1/2*. The results showed that when *psh1-1/2* was planted under a high exogenous nitrogen concentration condition (0.4 g N/kg soil), the shapes of grain, palea, and lemma in *psh1-1/2* were very similar to those of wild type, suggesting that the phenotype of *psh1-1/2* was partially recovered by exogenous nitrogen (Figure S2). These results confirmed the function of *OsARG* and validated the results of Ma et al. [42].

In conclusion, *psh1-1/2* are two new mutants of *OsARG*. They showed some similar mutant phenotypes to those of the reported mutant *nglf-1*, but also displayed some distinct mutant phenotypes, especially on the shapes of floret and seed, probably because of the influence of the genetic background, suggesting that *OsARG* may function differently under different genetic backgrounds. The results of this study will help better understand the function of *OsARG* and provide more information for further study on the growth and development of rice flowers.

## Figures and Tables

**Table 1:** Phenotypic analysis of WT (Minghui 86) and *psh1*.

Trait	Minghui 86	*psh1*
Plant Height (cm)	11.6 ± 0.91	11.2 ± 0.95
Tiller Number	7.6 ± 1.71	6.1 ± 3.07
Panicle Length (cm)	3.0 ± 0.13	2.5 ± 0.19 *
Spikelet Number per Panicle	199.7 ± 21.28	181.9 ± 30.03 *
Primary Branch Number	12.3 ± 0.95	11.9 ± 1.45
Secondary Branch Number	48.9 ± 7.31	46.0 ± 6.57
Seed Setting Rate (%)	85.2 ± 4.98	5.8 ± 7.28 **
Grain Length (mm)	9.4 ± 0.66	8.7 ± 0.48 *
Grain Width (mm)	2.8 ± 0.42	2.0 ± 0.08 *
1000-Grain Weight (g)	28.7 ± 0.66	15.0 ± 1.19 **

Note: Values are mean ± SD. Asterisks indicate significant differences to wild type (MH86) at *p* < 0.05 (*) or *p* < 0.01 (**) according to *t*-test (*n* = 10).

## Data Availability

Not applicable.

## Supplementary Materials

The following are available online at https://www.mdpi.com/article/10.3390/life11060523/s1, Figure S1: Cross-section of flowers of *psh1* and wild type by resin section, Figure S2: Grain shapes of *psh1* under low and high exogenous nitrogen concentration.
