# Supplementary material for: Loss of Function of OsARG Resulted in Pepper-Shaped Husk in Indica Rice
Source: Life (Basel). 2021 Jun 3;11(6):523. doi: 10.3390/life11060523 (PMC8227114; doi:10.3390/life11060523)
Supplement: Supplementary file 1 [file life-11-00523-s001.zip › life-1199913-supplementary.pdf]

Supplementary Metarials

Loss of Function of *OsARG* Resulted in Pepper-Shaped Husk in *Indica* Rice

Yan Zheng [1,2,*], Mjomba Fredrick Mwamburi [1,2], Huaqing Liu [3] and Feng Wang [3]

[1] College of Life Sciences, Fujian Agriculture and Forestry University, Fuzhou 350002, China; fmjomba@tum.ac.ke
[2] Fujian Key laboratory of Crop Breeding by Design, Fujian Agriculture and Forestry University, Fuzhou 350002, China
[3] Institute of Biotechnology, Fujian Academy of Agricultural Sciences, Fuzhou 350003, China; lhq@fjage.org (H.L.); wf@fjage.org (F.W.)
* Correspondence: zhengyan@fafu.edu.cn; Tel.: +86-591-83789176

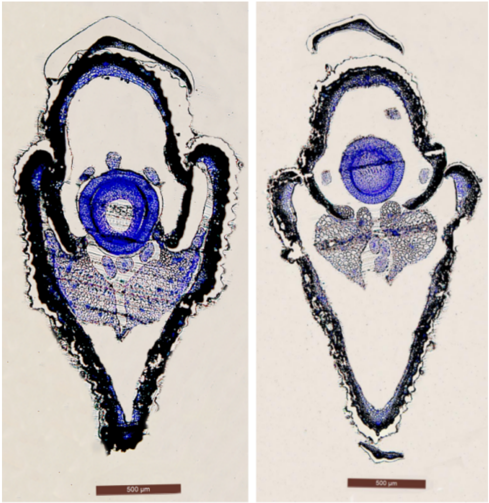

WT *psh1*

Figure S1. Cross section of flowers of wild type and *psh1* by resin section.

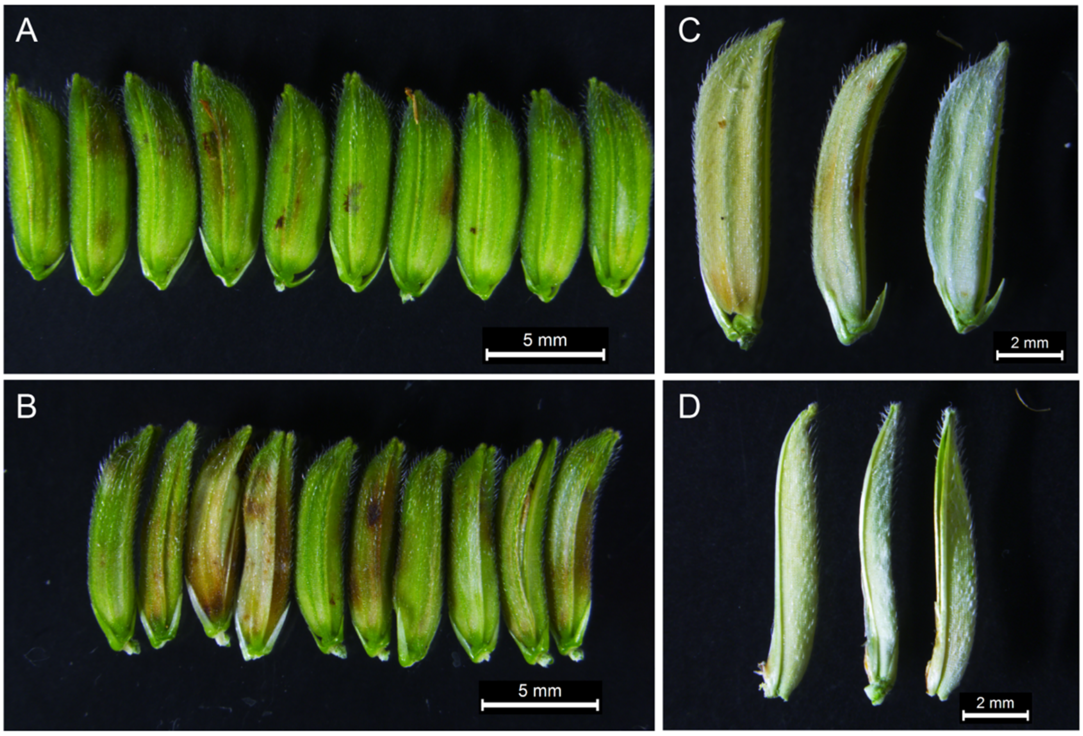

Figure S2. Grain shapes of *psh1* under low and high exogenous nitrogen concentration. (**A**) Grain shapes of *psh1* under 0.4g N/kg soil exogenous nitrogen concentration. (**B**) Grain shapes of *psh1* under 0.1g N/kg soil exogenous nitrogen concentration. Lemma (**C**) and palea (**D**) of *psh1* under 0.4g N/kg soil exogenous nitrogen concentration.
